# Dysmenorrhea1Read before the section on Obstetrics and Gynecology of the Buffalo Academy of Medicine, December 26, 1893.

**Published:** 1894-03

**Authors:** Electa B. Whipple

**Affiliations:** 491 Porter Avenue; Buffalo, N. Y.


### Original Communications.

### DYSMENORRHEA.[1]

By ELECTA B. WHIPPLE, A. M., M. D., Buffalo, N. Y.

WITH SOME hesitancy would the topic under consideration be brought before this body of learned, representative gynecologists, were it not true that disease and suffering, however trivial they may appear in the light of science, demand recognition and must ever receive attention from the conscientious physician. Hence, no apology is required for presenting a subject trite as its literature is meager and broad as its pathology is obscure.

In view of this dearth in its literature and the many and diverse theories held, the student must conclude that the etiology of dysmenorrhea is but imperfectly understood. In considering this important subject, whether in point of its etiology, prophylaxis, treatment, or citation of cases under observation, we would emphasize *excessive* dysmenorrhea in contra-distinction to the pain, aching and lassitude ordinarily attending the menstrual function.

It matters little to the suffering patient whether this affection be primary or secondary in character, or whether it be a symptom of some structural, pathological condition, or classed in the great family of neuroses. But, under a nomenclature, varying as the pathology is conceived to be, the physician is called upon to treat a neuralgic, inflammatory, congestive, membranous, obstructive, or, more properly, an ovarian, uterine, or a constitutional dys menorrhea.

### WHO ARE THE SUFFERERS?

This affection is not confined to the overworked, hysterical shop-girl, standing upon her feet nearly from "sun to sun," nor yet to the anemic, neurasthenic seamstress, who passes six days of every week at the other horn of the dilemma, and rarely rises from her chair in working hours. Nor can the society "bud," fritting her life away in late hours and rash exposures, be in this exclusive, even from the ambitious school-girl, striving to be the "star scholar" in her class, and thus furnishing a convenient target at which anti-educationists may aim their luckless shots. But the rosy-cheeked, romping, rollicking, carefree and easy

1. Read before the section on Obstetrics and Gynecology of the Buffalo Academy of Medicine, December 26, 1893.

country girl, well in every other respect, frequently suffers tortures at each monthly epoch.

Again, dysmenorrhea is not only frequently present in the sterile, but also in some *parous* women where no displacements exist, nor can any anatomical irregularities be discovered, either by microscopic or macroscopical examination.

### ETIOLOGY.

That it is most essential to understand the etiology of a disease before undertaking its treatment, goes without saying. Yet in the literature of dysmenorrhea we find eminent gynecologists averring that "the relations between dysmenorrhea and its causes are very diverse and imperfectly understood, that no single theory of its causation will apply in all cases, and that no nosological system covers the ground satisfactorily from a clinical point of view. Nevertheless, it is convenient to treat of the affection under some of the various forms that we have assigned to it."

(*a*) Ovarian: That dysmenorrhea may be due to inflamed or prolapsed ovaries is rational, especially when the excessive pain referred to that region occurs from one week to ten days previous, or following the menstrual epoch. Several cases of this kind come vividly to the mind of the writer, to two only of which will reference be made.

Case I.—M. C., unmarried, age about 24, blonde, came under observation for relief from periodical suffering. She was well developed, of fine physique, stately carriage, full of energy and activity and not given to "sedentary laziness." About one week previous to each menstruation she was seized with severe lancinating pains, commencing in right ovary and radiating through the entire pelvic region, until, as she expressed herself, she suffered from "unbearable cramps," which finally ceased and the menstrual period was passed in a most comfortable and painless manner.

Case II.—S. M., married, sterile, blonde, menstruation was unattended with suffering, but, about one week after the period, she invariably experienced intense and increasing pelvic pain, generally culminating in a prostrating headache.

(*b*) Uterine: What an array of young women and girls every year present themselves not only to the specialist, but to the general practitioner, for relief from distressing dysmenorrhea!

The etiology of many of these cases is confirmed by an examination, and under the proper treatment for congestions, displacements of the uterus and stenosis of the cervical canal, they are relieved.

But the greater part either have immunity for a considerable length of time, pass from the notice of the physician who considers them cured, and later the old trouble recurs, or they drift into the care of another, who under similar treatment may give some relief, or entirely fail in the attempt.

For illustration, a few instances of this kind may be mentioned:

Case I.—K. N., age 22, well developed, good color, bright, brown eyes, in good general health, active, vivacious, a school-teacher, complained of intense and increasing dysmenorrhea, so that she was compelled to lie in bed for two or three days at each menstrual period. Not only did she experience intense pain in back, hips and throughout pelvis, so that morphia was liberally used, but incipient rigors, temporary contraction of facial muscles, rigidity and loss of consciousness were present, when her physician resorted to the use of chloroform. Uterus was in nearly normal position, dilatation of cervical canal gave marked relief for a short time, but did not continue.

Case II.—M. E., unmarried, age 21, brunette, fine physique, plump, a stenographer, suffered from dysmenorrhea, uterus was in normal position, dilatation of cervical canal gave some improvement, but after a few months usual pain returned.

Case III.—M. S., unmarried, age 27, anemic, flow scanty, uterus somewhat retroverted, but no benefit was experienced from dilatation of cervix, which had been made by her physician.

Case IV.—M. W., unmarried, age 20, of a very nervous temperament, irritable, a stenographer. Each menstrual period was ushered in with great suffering, flow profuse, lasting five or six days. Uterus was retroverted but no discoverable stenosis, though her physician had resorted to dilatation, which operation furnished not the least relief.

At the sixth annual meeting of the American Association of Obstetricians and Gynecologists, held in June, 1893, in a most interesting discussion following the reading of a paper on Dilatation of the Cervix for Dysmenorrhea, one of the participants stated that from his investigations, extending over a period of ten years, where post-mortem examination of more than one thousand uteri had been made, the condition of stenosis of the uterine canal, in the lower segment, was one of the rarest conditions ever occurring in that organ.

And what shall we say of those women who suffer from endometritis, displacements and numerous pelvic diseases, and yet painful menstruation is unknown to them, while women having normal ovaries, tubes and uteri suffer tortures at each menstrual period?

This brings us within the domain of

(*c*) Constitutional dysmenorrhea: Under this classification may be found the anemic, chlorotic, neurasthenic, overworked and underworked girls—all generally most unhygienic in their personal habits.

Many of these are of healthy parentage, endowed with a heritage of robustness, and at the age of puberty possess every apparent perquisite of health. The menstrual function is established with but little pain, yet at each successive period becomes more pronounced and aggravated until in a few years the recurrence becomes well-nigh unbearable.

It is not surprising to find this condition in women clerks, standing all day upon their feet, laboring under more or less excitement, wearing their clothing uncomfortably tight, going to and from their work exposed to wet and cold, and evenings refraining from no pleasures that may conflict with a proper care of their health. And we are not astonished that sewing women, stenographers, book-keepers, and those of sedentary habits, taking little or no exercise in the open air, and denying themselves of sufficient, good nourishing food are generally sufferers from dysmenorrhea. Yet neither of these classes presenting themselves to the physician apparently receive one-half the solicitude that does the school-girl. And when the mother consults the family physician regarding any irregularities that menace the health of her fond daughter, as if a confession of inability to relieve, it is quite popular to advise *immediate removal from school.*

Prof. Gill Wylie, in an article published in the *American System of Obstetrics and Genecology*, Vol. I., says: "It is a well-recognized fact that dysmenorrhea is much more common among the highly educated and well-to-do classes than among the laboring classes." Further he says:

This is probably due to two causes: First, among the rich, the law of survival of the fittest is interfered with; second, besides being enfeebled by bad hygienic environments, the girls of the rich, as they approach puberty, are compelled to expend all available force in intellectual work at a time when the generative organs should be developed; even where a good constitution is inherited and sufficient exercise, food and sunlight are allowed to fairly well develop the muscular system, if emotional and intellectual work is forced upon them during the period that the generative organs should be developed, or allowed force to develop, they will be likely to suffer from dysmenorrhea due to imperfect development of the generative organs.

It has been asserted that the poor, "being exempt from the rigid laws and customs of civilization imposed upon the cultured and wealthy classes," are almost entirely free from local troubles. But such is not the case, as the charity clinics in our hospitals testify. And we know that many of the laboring women and girls who have spent very little time in educational pursuits, but having been subjected to prolonged bodily fatigue and heavy menial work, suffer greatly from pelvic ailments.

Much has been written upon this subject and some statistics have been gathered, and yet they are so few and incomplete that no logical conclusion can be deduced from them. No general, systematic research has been made to determine in what way physical growth and development, during the age of puberty, are influenced by the mental work imposed by our schools. It does not appear that the average American youth is in any immediate danger of becoming a physical wreck on account of the difficult curriculi of study in the common schools. When we consider that the average number of daily school hours for all the grades is only about five, with intermissions, and the study periods will not average more than one and one-half or two hours during the sessions, and but one or two hours are devoted to study out of school, it hardly seems plausible that intellectuality and culture are acquired by our girls at the expense of physical development. Dr. Mary Putnam Jacobi once pithily said:

> It is in fact a matter of common observation that hysterical and anemic women, in whom disordered menstruation is most frequently observed, are conspicuously destitute of habits implying either cerebral or spinal activity; that is, they neither think much, nor take much physical exercise.

The health statistics of women college graduates, reported by a special committee of the Association of Collegiate Alumnæ, together with statistical tables collated by the Massachusetts Bureau of Statistics of Labor in 1885, gave in its summary of results that the average age at which the girls began study was 5.64 years, at entering college 18.35 years, at graduating from college 22.39 years. Furthermore, that the female graduates of our colleges and universities do not seem to show, as the result of their college studies and duties, any marked difference in general health from the average health likely to be reported by an equal number of women engaged in other kinds of work, or, in fact, of women generally without regard to occupation followed. Why, then, the very diverse conclusions arrived at by those who have made but limited observations for special purposes? The small amount of mental work imposed by the schools cannot in itself be deleterious, but the evil lies in the methods of accomplishment. The school-girl cannot be a society girl and maintain an ideal standard of health. School work, supplemented by late hours, midnight banquets, heating and suddenly cooling the surface of the body, tight clothing, and going out of doors with feet incased in paper-soled slippers, surely are not contributory to health and do swell the statistics.

Neither can the school-girl, with impunity, lead a passive life and, because of her preference, remain indoors during the school intermissions, doing the most desultory studying, eating confectionery, or an indigestible baker's lunch, when she should be in the open air engaged in unrestrained exercise.

We concur with Prof. Wylie and others, that a great source of dysmenorrhea is imperfect development of the generative organs, which, however, we believe is due, not so much to the force expended in intellectual work, but rather to a lack of systematic, hygienic exercise and a profound ignorance of the anatomy and physiology of the pelvic and other organs of the human body. How to remedy this alarmingly growing evil, and thus avert a vast amount of suffering with its disastrous sequelæ, is a problem requiring for its solution the thought of every progressive physician. And this brings us to the very threshold of

PROPHYLAXIS, toward which we note the public trend. The keynote of prophylaxis was struck by Dr. Delancy Rochester, President of the Buffalo Academy of Medicine, in his scholarly and timely address before that body, last June. Said he:

> In our system of school education should be included an elementary course in human physiology, in which every child should be instructed before he or she has reached the age of fourteen or fifteen years. The instructor in physiology should always be a thoroughly educated physician.

This sentiment we most heartily indorse, and, furthermore, we believe that daily hygienic exercise should be provided for every pupil in our schools, under the direction of an educated physician. Then will the aphorism of Emerson no longer be forcibly true: "Society never advances; it recedes as fast on one side as it gains on the other." Then the intellectual will not be gained at the expense of the physical. Surely it is significant of this progressive age when we find the public educator in unison with the physician and working not only along lines but also in a physiological manner, and directing the *manual* as well as the mental and moral training—the highest development of all being the only *true* education.

We believe we are correct in the statement that in all the grades of our city schools a smattering of physiology is now taught, and in the High School, we are pleased to say, physiology has for some time been taught by a physician. Also, in some of the grades, at least, a feeble attempt is made to teach physical culture, and we gladly note even these initial advances along the lines of progressive thought and education. If the law for compulsory education can be created and enforced, why not a law for compulsory hygienic exercise in the schools?

The family physician may be able to do something towards impressing the mother as to her duty in imparting to her offspring whatever knowledge of physiology and hygiene she may possess. But, in the majority of cases, there is a woeful ignorance, coupled with that unaccountable diffidence existing between mother and daughter in regard to those physiological subjects, where the greatest frankness should be maintained. As a result, dysmenorrhea and other pelvic disorders are inaugurated, which the girl may never overcome. For these and obvious reasons, we believe special instruction in our schools should be given by one thoroughly competent, when greater heed will be taken and the health interests of those instructed be better subserved.

Also, the daily hygienic exercise, whether from the Swedish, Sargent, Emerson, or Delsarte systems, should be given under the direction of a physician. Then will each pupil receive, at the outset, a physical examination, measurements be taken, and strength tested, which tests, from time to time, will be repeated. Directions can then be carried out in regard to the dress, and, at least, five days in the week will the hygienic dress, suitable for engaging in free exercise, be worn. Each will take the exercise suited to her condition, and not only be taught to sit, to stand, to walk, and to carry herself correctly, but the abdominal and pelvic muscles will receive their share of attention and, we believe, the great *desideratum*—proper development of the generative organs—will be better attained.

TREATMENT.

Since the purpose of this paper is the consideration of the etiology and prophylaxis, rather than the treatment, of dysmenorrhea, but brief reference will be made to the general therapeutics.

The suggestion of a vaginal examination is a great shock to the sensitive, shrinking nature of the young girl, worn out with suffering at each menstrual period, and terrified at the expectancy of a recurring one. Nor will the considerate physician exact this until all probable and improbable remedies have been exhausted, without success, in the endeavor to overcome the persistent dysmenorrhea. If the sufferer is then unrelieved, more radical measures must be employed.

But the first step in the treatment of the married woman should be a thorough examination for the purpose of detecting any existing displacements, stenosis of the cervical canal, or congestion of the generative organs.

If displacements, flexions, stenoses, occlusions or polypi be present, and in some diseased conditions of the ovaries, only operative measures will give relief.

In many cases due to inflammation of uterus and ovaries the use of galvanism has given great satisfaction in completely overcoming the existing dysmenorrhea.

If the patient be anemic or chlorotic, a tonic course of treatment, with enforcement of strict hygiene, is indicated.

The habit which some young women and girls have formed of using alcohol and morphia—at first for relief from dysmenorrhea, and finally for every ache and pain until they find the habit too firmly fixed to be discontinued—should be denounced in no uncertain terms by the family physician.

In an abstract of a paper by Thomas More Madden, M. D., read before the International Medical Congress, Berlin, August, 1890, he says:

Of all the ailments of female existence, few give rise to more persistent suffering, or produce more disastrous effects on the general health and even on the cerebro-nervous system, or on the moral constitution of the patient than does well marked, obstructive dysmenorrhea. The latter consequence is more especially evident in many cases of alcoholism, which, in women, may frequently be dated from their first painful menstrual period, for the relief of which stimulants are too often improperly administered and repeated in increasing doses until finally, in many cases, the victim of dysmenorrheal alcoholism becomes an habitual and perhaps incurable drunkard.

In those cases characterized by a scanty menstruation, in which no well-defined local or organic lesion can be found, but where a neurotic or hysterical tendency prevails, the employment of drugs generally proves successful.

But a proper selection of remedies can be made only after a careful study of each individual case, since that agent which brings relief to one from distressing dysmenorrhea may be utterly worthless for another, and what proves a success at one time may be a failure at the next.

491 PORTER AVENUE.

